# Supplementary material for: MetaBAT 2: an adaptive binning algorithm for robust and efficient genome reconstruction from metagenome assemblies
Source: PeerJ. 2019 Jul 26;7:e7359. doi: 10.7717/peerj.7359 (PMC6662567; doi:10.7717/peerj.7359)
Supplement: Supplemental Information 1 — IMG access IDs and Sample names for IMG-100 dataset. [file peerj-07-7359-s001.pdf]

Table S1: A list of metagenome assemblies used to evaluate binning performance

AssessionID	Sample Name
1000607	Alkali sediment microbial communities from Soda Lake, CA
1010336	Marine cyanobacterial communities from Panama
1021939	Serpentinite rock and fluid subsurface biosphere microbial
1041346	Freshwater lake sediment microbial communities from the University
1049274	Marine gutless worms symbiont microbial communities from Max Planck
1061688	Cellulose-adapted microbial communities from the Joint BioEnergy
1064129	Surface soil microbial communities from Centralia Pennsylvania
1091231	Freshwater microbial communities from Lake Fryxell liftoff mats
1104152	Peatland microbial communities from Minnesota, USA, analyzing carbon
1000652	Forest soil microbial communities from El Dorado National
1013404	Cellulose adapted compost microbial communities from Newby
1021953	Olavius algarvensis symbiont microbial communities from Tuscany
1045045	FECB-22 metaG
1049298	Marine gutless worms symbiont microbial communities from Max Planck
1061692	Cellulose-adapted microbial communities from the Joint BioEnergy
1064219	Warmed soil microbial communities from the Hubbard Brook experimental
1091232	Freshwater microbial communities from Lake Bonney liftoff mats
1104156	Peatland microbial communities from Minnesota, USA, analyzing carbon
1001465	Subsurface groundwater microbial communities from S. Glens
1015567	Wetland sediment microbial communities from Twitchell Island
1021962	Olavius algarvensis symbiont microbial communities from Tuscany
1045048	FECB-24 metaG
1049301	Marine gutless worms symbiont microbial communities from Max Planck
1061693	Cellulose-adapted microbial communities from the Joint BioEnergy
1066080	Iron sulfur acid spring bacterial and archeal communities from Banff
1091682	Rumen microbial communities from New Zealand - Rumen Methanol Enrichment
1104464	Active sludge microbial communities of municipal wastewater-treating
1001468	Subsurface groundwater microbial communities from S. Glens
1016497	Nasutitermes corniger P1 segment microbial communities
1022553	Hot spring thermophilic microbial communities from Obsidian
1045051	FECB-27 metaG
1049376	Wastewater treatment Type I Accumulibacter community
1061709	Cellulose-adapted microbial communities from the Joint BioEnergy
1066081	Hot spring sediment bacterial and archeal communities from British
1092161	Estuarine microbial communities from the Columbia River estuary
1104465	Active sludge microbial communities of municipal wastewater-treating
1001471	Subsurface groundwater microbial communities from S. Glens
1016500	Nasutitermes corniger P3 segment microbial communities
1024027	Soil microbial communities from Rifle Colorado, Rifle Oxygen_injection
1045180	Worm MetaG Olavius albidus HERON ISLAND.1
1049379	Wastewater treatment Type I Accumulibacter community
1061719	Anoxygenic and chlorotrophic microbial mat microbial communities

1066082 Hot spring sediment bacterial and archeal communities
1092165 Estuarine microbial communities from the Columbia River estuary
1104467 Active sludge microbial communities of municipal wastewater-treating
1004322 Freshwater microbial communities from Lake Mendota WI
1016600 Arctic peat soil from Barrow, Alaska - NGEE Surface sample
1024981 Host-associated microbial community of the marine sponge
1045192 Worm MetaG Olavius imperfectus BELIZE.2
1049388 Wastewater treatment Type I Accumulibacter community
1061840 Enrichment culture microbial communities from Arthur Kill intertidal
1066083 Hot spring sediment bacterial and archeal communities
1092198 Estuarine microbial communities from the Columbia River estuary
1104480 Active sludge microbial communities of municipal wastewater-treating
1004517 Marine microbial communities from the Deep Pacific Ocean
1017313 Freshwater microbial communities from Lake Mendota, WI
1024987 Host-associated microbial community of the marine sponge
1045228 Worm MetaG Olavius ullae BAHAMAS.2
1049391 Wastewater treatment Type I Accumulibacter community
1061841 Enrichment culture microbial communities om Arthur Kill intertidal
1066084 Hot spring sediment bacterial and archeal communities
1092933 Freshwater sediment microbial communities from Prairie Pothole Lake
1104574 Hot spring microbial communities from South Africa to study Microbial
1004535 Marine microbial communities from the Deep Pacific Ocean
1017775 Hypersaline microbial mat communities from Conch Spring
1025707 Hot spring thermophilic microbial communities from Obsidian
1045249 Worm MetaG Olavius geniculatus LIZARD ISLAND
1049394 Wastewater treatment Type I Accumulibacter community
1061842 Enrichment culture microbial communities from rom New York Harbor
1066085 Hot spring sediment bacterial and archeal communities from British
1092937 Freshwater sediment microbial communities from Prairie Pothole Lake
1105275 Peat soil microbial communities from Weissenstadt, Germany - Sb_50d_b_LC
1004649 Marine microbial communities from the Deep Atlantic Ocean
1017781 Hypersaline microbial mat communities from Yellowstone National
1030626 Marine viral communities from the Pacific Ocean - ETNP_2_1000
1046603 Crystal Geyser 4/9/14 3 um filter
1049403 Wastewater treatment Type I Accumulibacter community
1062777 Switchgrass rhizosphere microbial communities from Kellogg Biological
1066086 Hot spring sediment bacterial and archeal communities from British
1093094 Active sludge microbial communities from wastewater in Klosterneuburg
1105508 Anaerobic biogas reactor microbial communites from Washington, USA
1009587 Aerobic enrichment media from Bioluminiscent Bay, La Parguera
1019791 Biofuel bioreactor microbial communities from Berkeley, California
1033754 Grasslands soil microbial communities from the Angelo Coastal
1047896 Wetland microbial communities from the San Francisco Bay
1056061 Freshwater sediment methanotrophic microbial communities
1062779 Switchgrass rhizosphere microbial communities from Kellogg Biological

1070358 Saline lake microbial communities from Ace Lake, Anatarctica-Antarctic
1093154 Extremophilic microbial mat communities from Yellowstone National
1009602 Aerobic enrichment media from Bioluminiscent Bay, La Parguera
1020040 Marine viral communities from the Deep Pacific Ocean - MSP-121
1035183 Freshwater microbial communities from Crystal Bog, Wisconsin
1047944 Wetland microbial communities from San Francisco Bay, California
1056139 Freshwater sediment methanotrophic microbial communities
1063486 Switchgrass rhizosphere microbial communities from Kellogg Biological
1073292 Marine eukaryotic communities from Monterey Bay, USA
1093505 Marine viral communities from the Global Malaspina Expedition
1009614 Tt an M2 anaerobic enrichment on miscanthus from sediment
1020097 Marine viral communities from the Pacific Ocean - LP-38
1035423 Freshwater microbial communities from Crystal Bog, Wisconsin, USA
1047947 Wetland microbial communities from San Francisco Bay, California
1056184 Freshwater sediment methanotrophic microbial communities
1064126 Surface soil microbial communities from Centralia Pennsylvania
1074340 Aqueous microbial communities from the Delaware River and Bay
1095630 Groundwater sediment microbial communities from an aquifer
1010297 Tt ae E1 feedstock associated aerobic enrichment on eucalyptus
1021909 Serpentinite rock and fluid subsurface biosphere microbial
1035438 Freshwater microbial communities from Crystal Bog, Wisconsin
1048537 Arabidopsis plate scrape CL_Col_mLB_r2
1056202 Freshwater sediment methanotrophic microbial communities
1064127 Surface soil microbial communities from Centralia Pennsylvania
1074528 Arctic peat soil microbial communities from the Barrow Environmental
1104138 Peatland microbial communities from Minnesota, USA, analyzing carbon
1010309 Tt ae P1 feedstock associated aerobic enrichment on pine
1021936 Serpentinite rock and fluid subsurface biosphere microbial
1039713 Marine microbial communities from expanding oxygen minimum
1048555 Arabidopsis plate scrape CL_Cvi_mLB_r2
1060143 Cellulose-adapted microbial communities from the Joint BioEnergy
1064128 Surface soil microbial communities from Centralia Pennsylvania
1091229 Freshwater microbial communities from Lake Fryxell liftoff mats
1104146 Peatland microbial communities from Minnesota, USA, analyzing carbon
