# Supplementary material for: MetaBAT 2: an adaptive binning algorithm for robust and efficient genome reconstruction from metagenome assemblies
Source: PeerJ. 2019 Jul 26;7:e7359. doi: 10.7717/peerj.7359 (PMC6662567; doi:10.7717/peerj.7359)
Supplement: Supplemental Information 2 — The parameter sets and their performance comparison to the default parameter set. [file peerj-07-7359-s002.pdf]

Table S2: List of parameter sets to evaluate MetaBAT2 on 120 real metagenome assem

Parameter Set	Max Edges	MaxP	MinS	Better	Same	Worse
**S1**	2000	91	60	32	75	13
**S2**	400	91	50	31	74	15
**S3**	500	91	80	31	75	14
**S4**	200	91	60	31	77	12
**S5**	200	98	60	13	88	19
**S6**	2000	95	60	7	105	8
**S7**	10000	95	60	6	106	8
**S8**	400	95	60	5	107	8
**S9**	3000	95	60	5	107	8
**Default**	200	95	60			

nblies

Total
120
120
120
120
120
120
120
120
120
